# Co-Expression of Host and Viral MicroRNAs in Porcine Dendritic Cells Infected by the Pseudorabies Virus

**DOI:** 10.1371/journal.pone.0017374

**Published:** 2011-03-08

**Authors:** Anna Anselmo, Laurence Flori, Florence Jaffrezic, Teresa Rutigliano, Maria Cecere, Naima Cortes-Perez, François Lefèvre, Claire Rogel-Gaillard, Elisabetta Giuffra

**Affiliations:** 1 Parco Tecnologico Padano, Polo Universitario, Lodi, Italy; 2 INRA, UMR 1313 de Génétique Animale et Biologie Intégrative, Jouy-en-Josas, France; 3 AgroParisTech, UMR 1313 de Génétique Animale et Biologie Intégrative, Jouy-en-Josas, France; 4 INRA, UR 892 de Virologie et Immunologie Moléculaires, Jouy-en-Josas, France; 5 CEA, DSV, IRCM, SREIT, Laboratoire de Radiobiologie et Etude du Génome, Jouy-en-Josas, France; The Centre for Research and Technology, Hellas, Greece

## Abstract

MicroRNAs are small non-coding RNAs approximately 22 nt long that modulate gene expression in animals and plants. It has been recently demonstrated that herpesviruses encode miRNAs to control the post-transcriptional regulation of expression from their own genomes and possibly that of their host, thus adding an additional layer of complexity to the physiological cross-talk between host and pathogen. The present study focussed on the interactions between porcine dendritic cells (DCs) and the Pseudorabies virus (PRV), an alpha-herpesvirus causing Aujeszky's disease in pigs. A catalogue of porcine and viral miRNAs, expressed eight hours post-infection, was established by deep sequencing. An average of 2 million reads per sample with a size of 21–24 nucleotides was obtained from six libraries representing three biological replicates of infected and mock-infected DCs. Almost 95% of reads mapped to the draft pig genome sequence and pig miRNAs previously annotated in dedicated databases were detected by sequence alignment. *In silico* prediction allowed the identification of unknown porcine as well as of five miRNAs transcribed by the Large Latency Transcript (LLT) of PRV. The gene target prediction of the viral miRNAs and the Ingenuity Pathway Analysis of differentially expressed pig miRNAs were conducted to contextualize the identified small RNA molecules and functionally characterize their involvement in the post-transcriptional regulation of gene expression. The results support a role for PRV miRNAs in the maintenance of the host cell latency state through the down-regulation of immediate-early viral genes which is similar to other herpesviruses. The differentially expressed swine miRNAs identified a unique network of target genes with highly significant functions in the development and function of the nervous system and in infectious mechanisms, suggesting that the modulation of both host and viral miRNAs is necessary for the establishment of PRV latency.

## Introduction

MicroRNAs (miRNAs) are small molecules (21–24 nucleotides long) involved in the post-transcriptional regulation of gene expression. They can inhibit the expression of specific messenger RNAs by binding to complementary target sequences located in the 3′ untranslated region (3′UTR) [1]. MiRNAs are expressed by all plants and animals, as well as by several DNA viruses. In particular, numerous members of the herpes virus family have been found to express several viral miRNAs in infected cells [2]. Once transcribed as precursors from the viral genome, these miRNAs are processed by the host and target both viral and host messenger RNAs. MiRNAs have several features that make them particularly useful to viruses: miRNAs are small and the evolution of a miRNA which is complementary to a new target gene can occur more easily than the evolution of a novel regulatory protein [3]. Moreover, miRNAs are not antigenic and the down-regulation of specific genes allows the virus to establish a favourable environment for its own replication while attenuating or avoiding the host immune response [3].

A particular feature of herpes viruses is their ability to establish and maintain latent infections wherein the virus genome circularizes and persists as an episome. In this state a limited set of viral transcripts, the latency transcripts, are expressed from specific regions of the genome, and the latent genome is most probably controlled at the epigenetic level [4], [5]. Several herpes viruses, such as Herpes Simplex Virus 1 (HSV1), Herpes Simplex Virus 2 (HSV2), Bovine Herpesvirus 1 (BoHV-1) and Epstein-Barr Virus (EBV) as well as other herpes viruses and large genome DNA viruses, contain miRNAs that regulate their own cycle [6], [7]. The miRNAs expressed by the latency associated transcripts have been related with the ability to establish long-term latent infections [8], [9], [10]. It has been demonstrated that the genomic positions of some miRNAs encoded by HSV-1 and HSV-2 are within or proximal to the Large Associated Transcript (LAT) [11]. In mouse cells infected by HSV-1, LAT functions as a primary miRNA precursor that encodes four distinct miRNAs [12]. Recently, deep sequencing studies have established comprehensive catalogues of the miRNAs expressed by human herpes viruses in latently infected cells of the human trigeminal ganglia [13], [14].

The Pseudorabies virus (PRV) is a porcine virus classified as a member of the Alphaherpesvirinae subfamily of Herpesviridae. PRV is the aetiological agent of Aujeszky's disease causing neurological, respiratory and reproductive disease in the pig, its' natural host. The genome of PRV is more than 142 Kb and is characterized by the presence of 70 different genes including the LLT, which for PRV is referred to as large latency transcript (LLT), and which is involved in maintaining the latency of PRV [15]. Recently host-pathogen interactions have been studied at the level of the transcriptome which showed that the gene expression of both PRV and porcine cells can be analyzed simultaneously using microarrays, thus providing a chronology of both PRV and host cell gene transcription [16]. PRV has been shown to be a pertinent model for the time-course transcriptomic study of herpes viruses and their mode of interaction with the host [17], [18]. The expression of most viral genes increases during infection, and many biological processes are altered during PRV infection [16]. However, no miRNA has been annotated for PRV and no data on host specific miRNA expression has been reported so far.

Dendritic cells (DCs) are the first immune cells to encounter pathogens during infections. They function as crucial mediators linking innate and adaptive immunity through their ability to synthesize cytokines and to act as professional antigen presenting cells to activate naïve T cells [19], [20]. DCs are known to play a key role in initiating immune response to viral infection and many viruses have evolved specific evasion strategies to suppress host immune responses via interaction with DCs [21], [22]. The porcine blood is very similar to human blood in representing the source of DC precursors, and porcine immature DCs can be differentiated *in vitro* from peripheral blood monocytes [23]. PRV has been shown to infect this cell system efficiently [Naima Cortes-Perez and François Lefèvre, manuscript in preparation]. Therefore, porcine DCs represent a good model to evaluate the role of both viral and cellular miRNAs in terms of host response to PRV's infection.

The aim of this study was to characterise the miRNA regulation layer taking place in porcine DCs in the course of PRV infection using a deep sequencing approach.

## Results

### Biological model

Pig DCs were obtained by *in vitro* differentiation of peripheral blood monocytes from three Large White pigs. The DCs were either infected with PRV or mock-infected, in triplicate. A total of three replicates were infected using a PRV virulent strain (NIA-3) at a multiplicity of infection (MOI) of 3. Cells were harvested for miRNA library preparations at 8 hours post-infection (PI) as a large increase in viral gene expression is already seen in DCs at 4 h PI which coincides with the first viral replication, whereas by 12 h PI most viral genes are differentially expressed [16].

### Mapping of sequence tags to the pig and PRV genomes

Six small RNA libraries were produced from the DC samples and analysed by deep sequencing (Solexa/Illumina technology) which produced, on average, 2.7 million reads per sample. After the trimming of adapters, all the inserts from 21 to 24 bases were mapped onto the pig and PRV genomes with the MAQ software [24]. Overall 96% of inserts with a minimum mapping quality of 10 were mapped on either the pig genome sequence (Build 8) or the PRV genome, and 88.9% of the reads could be aligned with a mapping quality of 40 or higher. For all samples, over 83% of the inserts (ranging from 83.6% to 86.9%) were mapped without errors; only a small fraction of the inserts were attributed to repeat regions (2.7–6.3%). Infected DCs showed a consistent lower number of sequence reads compared with mock infected cells, in the order of 18% (animal 1), 30% (animal 2) and 25% (animal 3). The vast majority of mapped sequence tags originated from the pig genome and only a small fraction from the PRV genome. A small background signal of sequences that mapped to the PRV was observed in mock infected DCs, approximately 0.5% (animals 1 and 2) and 1.5% (animal 3). On average, 923 sequences were exclusively expressed by the virus, with 83% of the viral sequences being in common among the three infected samples (Table 1).

**Table 1:** Mapping of sequence reads on the Pig and PRV genomes.

	Animal 1		Animal 2		Animal 3	
	GDI3	GDI4	GDI5	GDI6	GDI7	GDI8
**Inserts (21–24nt)**	1781519	1467701	2112969	1530897	2238837	1664479
**q10 mapped inserts**	1704905	1403824	2036278	1425408	2147317	1612865
**% of q10 mapped inserts**	96%	95.6%	96.4%	93.1%	95.9%	96.9%
**q10 inserts on ** ***S. scrofa***	1704900	1402857	2036274	1424676	2147301	1611795
**q10 inserts on PRV**	5	967	4	732	16	1070

Number of reads obtained for each sequenced sample of DCs (mock-infected: GDI3, GDI5, GDI7; infected by PRV: GDI4, GDI6, GDI8), percentages of reads mapping on the PRV and *Sus scrofa* genomes using the MAQ alignment software, and number of reads aligned with the minimum mapping quality of 10.

### Identification and mapping of miRNAs expressed by PRV

The alignment of sequencing reads expressed by the PRV with miRNAs known in other herpesviruses did not provide any significant match, in agreement with previous reports of low conservation of miRNA sequences among viruses [9], [25].

**Figure 1:**
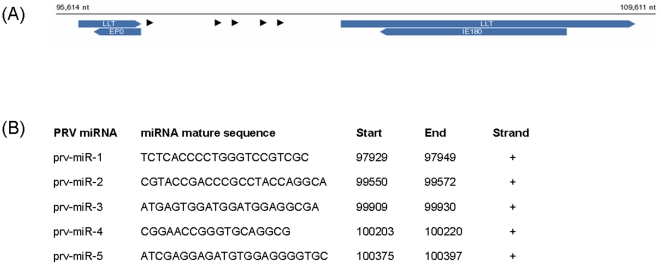
miRNA and LLT. Genomic location (A) and genomic coordinates (B) of five miRNAs predicted in the PRV genome. The five miRNAs are contained in the intronic region of the LLT transcript. Genomic coordinates refer to the PRV genome (Entrez: NC_006151). Arrows point to miRNAs position on the PRV sequence.

A discovery analysis was carried out with the software miRDeep [26] and led to the identification of four new putative PRV miRNAs ranging between 21–23 nt. Moreover, miRDeep identified an additional 18 nt sequence as a mature miRNA (Figure 1). The genomic locations of these five miRNAs (prv-miR-1 to prv-miR-5) indicated that they are transcribed as part of the intronic region of the Large Latency Transcript (LLT), more precisely from positions 97929 to 100397 of the viral genome. The genomic regions encompassing the novel PRV miRNAs were each found to fold into energetically stable hairpin structures (Figure S1).

Sequence heterogeneity was found for most sequence tags when compared to the mature miRNAs. In particular, 3′ heterogeneity was more commonly found than 5′ heterogeneity. This is in keeping with findings from other miRNA deep sequencing studies and with the finding that most of the variability occurs in either Dicer1 or Drosha cleavage positions within the pre-miRNA hairpin [27], [28]. Excluding miR-2, for which a single mature miRNA was predicted, the other miRNAs corresponded to the most abundant sequence tags aligning with the viral genome (Table 2).

**Table 2:** Prediction of mature PRV miRNAs in infected porcine DCs.

PRV miRNA	Sequence	Length (nt)	# reads	frequency (#reads/tot #reads)
**prv-miR-1**	TCTCACCCCTGGGTCCGTCGC	21	515	0.1859
	TCTCACCCCTGGGTCCGTCGCTA	23	1	0.0004
	TCTCACCCCTGGGTCCGTCGCT	22	19	0.0069
	TCTCACCCCTGGGTCCGTCGCCA	23	1	0.0004
	TCTCACCCCTGGGTCCGTCGCC	22	5	0.0018
	TCTCACCCCTGGGTCCGTCGCAC	23	1	0.0004
	TCTCACCCCTGGGTCCGTCGCAA	23	4	0.0014
	TCTCACCCCTGGGTCCGTCGCA	22	5	0.0018
	CTCTCACCCCTGGGTCCGTCGC	22	1	0.0004
	ATCTCACCCCTGGGTCCGTCGC	22	2	0.0007
**prv-miR-2**	CGTACCGACCCGCCTACCAGGCA	23	8	0.0029
**prv-miR-3**	ATGAGTGGATGGATGGAGGCGA	22	232	0.0838
	ATGAGTGGATGGATGGAGGCGAAA	24	6	0.0022
	ATGAGTGGATGGATGGAGGCGAG	23	4	0.0014
	ATGAGTGGATGGATGGAGGCGAA	23	19	0.0069
	ATGAGTGGATGGATGGAGGCGAGA	24	4	0.0014
	ATGAGTGGATGGATGGAGGCGAGT	24	3	0.0011
	ATGAGTGGATGGATGGAGGCGAAT	24	2	0.0007
**prv-miR-4**	CGGAACCGGGTGCAGGCG	18	450	0.1625
	CCGGAACCGGGTGCAGGCG	19	77	0.0278
	GCCGGAACCGGGTGCAGGCG	20	12	0.0043
	CGCCGGAACCGGGTGCAGGCG	21	3	0.0011
**prv-miR-5**	ATCGAGGAGATGTGGAGGGGTGC	23	553	0.1997
	ATCGAGGAGATGTGGAGGGGTGCC	24	5	0.0018
	ATCGAGGAGATGTGGAGGGGTGCA	24	414	0.1495
	ATCGAGGAGATGTGGAGGGGTGCT	24	167	0.0603
	ATCGAGGAGATGTGGAGGGGTGCG	24	18	0.0065

For each miRNA sequence, nucleotide sequence, sequence length and number of sequence reads (#) and frequency are reported. The most abundant sequence among the hypothetical miRNAs was considered as the most likely mature miRNA.

### Gene target analysis of viral miRNAs

The 70 annotated viral genes were analysed to identify putative targets for the five predicted viral miRNAs (prv-miR 1, 2, 3, 4, 5). The complementarity of each miR 5′ seed sequence, the low free energy and the conservation of the target site were considered as main features for target mRNA discovery. The most significant targets genes were identified by miRanda [29] and verified by TargetScan [30] and PITA [31]. Seven out of eleven targets were confirmed with significant scores by both TargetScan and PITA, while three of them were only confirmed by PITA and one was not confirmed (Table 3). Possible targets for prv-miR-1-5 include the LLT itself and the viral genes UL28 (Unique Long region 28), UL33, UL34, UL43, UL47, UL48, US1, EP0 (Early Protein 0) and IE180 (Immediate Early Protein 180). UL28 and UL33 are involved in DNA cleavage and packaging, UL34 and UL47 are involved in viral egress (nuclear egress and secondary envelopment respectively), whereas UL48, EP0 and IE180 are involved in gene regulation as transactivators of transcription [15].

**Table 3:** Gene target analysis of PRV miRNAs.

				miRanda		TargetScan	PITA
Target gene	Coordinates	strand	miRNA	score	energy	Site_type	ΔΔG score
EP0	96247:97736	-	prv-miR-3	154	−23.79	m8	−16.14
IE180	102696:107804	-	prv-miR-5	181	−38.68	m8	−24.29
LLT	96079:109118	+	prv-miR-2	148	−32.15	**-**	−12.93
LLT	96079:109118	+	prv-miR-3	148	−30.09	m8	−22.86
UL28	16808:21741	-	prv-miR-5	169	−30.70	m8	−15.83
UL33/UL34	30892:32571	+	prv-miR-2	147	−22.62	**-**	**-**
UL43	52798:53988	+	prv-miR-4	163	−31.88	m8	−21.48
UL47	11695:16176	+	prv-miR-1	167	−32.12	m8	−23.85
UL48	10332:16176	+	prv-miR-1	167	−32.12	m8	−23.85
US1; SuHV1_gp64	115211:117215	+	prv-miR-4	152	−28.62	**-**	−17.63
US1; SuHV1_gb73	127387:129391	-	prv-miR-4	152	−28.62	**-**	−17.63

The two top PRV target genes predicted by the software miRanda [29] are listed for each viral miRNAs. Genomic coordinates, score and energy (kcal/mol) are presented as indicated by the software. TargetScan [30] and PITA [31] were used to confirm the predicted target genes. For TargetScan, m8 indicates an exact match of the seed to positions 2–8 of the mature miRNA (the seed + position 8), while the ΔΔG score reported for PITA is an energetic score. Dashes indicate absence of significant prediction.

### Identification of known and novel porcine miRNAs

As for the viral tags, small RNA sequences expressed by *Sus scrofa* were first grouped to identify identical reads and to define the counts associated with particular sequence tags. These non-redundant reads were then mapped against all the miRNAs annotated in miRBase (release 16) [32].

Of the 211 known porcine miRNAs (ssc-miRNAs) perfect matches were found with an average of 96.5 entries per sample and a total of 156 pig miRNAs were identified (Table S1).

An additional set of 248 porcine sequence tags were identified as miRNAs using a comparative approach (Table S2). The number of porcine miRNAs currently annotated in miRBase is low compared to other higher eukaryotes (i.e. only 211 miRNAs annotated in the pig compared to 1048 in *Homo sapiens*). Despite the existence of lineage-specific miRNAs (e.g. the primate-specific miR-650 cluster [33] a large number of miRNAs is evolutionary conserved; it is thus expected that a similar number of miRNAs would be present in all mammalian species. Sequence tags were aligned with miRNAs annotated in miRBase for *Homo sapiens*, *Mus musculus*, *Bos taurus*, *Canis familiaris*, *Equus caballus* and *Gallus gallus*. Only the reads showing a perfect match with the mature miRNA in these other mammals were defined as orthologous porcine miRNAs. The pig origin of 115 orthologous miRNA was confirmed by alignment of each specific pre-miRNAs to the pig genome (Table S2).

In addition, a prediction of putative novel miRNAs was carried out using the whole set of sequencing data. Using the sequence tags position on the genome and secondary structure folding energy, 27 sequence tags could be predicted as new pig miRNAs. Fifteen of the newly predicted miRNAs ranged between 21–24 nucleotides, ten ranged between 16–20 nt and three ranged between 25–26 nucleotides in length. Nine of the 27 newly predicted miRNAs were found in more than one sample and so they were considered as the most reliable (Table S6).

### Relative abundance of porcine miRNAs in DCs

The sequence counts revealed that levels of expression displayed a very large range, varying from single counts to several thousands reads for the most abundant miRNAs. However, most miRNAs were expressed at comparable levels in both control and infected DCs (Table S1). In particular miR-21 was the most abundantly expressed miRNA, with a mean of nearly 1,500,000 sequence found in both infected and control samples representing almost 91% of all small RNA sequence tags.

To verify that this high expression of miR-21 was not due to technical artefacts of deep sequencing, the expression of miR-21 was examined using qPCR in independent samples (DCs challenged with PRV in the same conditions and collected at 4 and 12 hours PI) and compared to the levels of expression of six known miRNAs (miR-339-3p, miR-184, miR-7, miR-370, miR-708*, miR-29b-1*) and U6 small nuclear RNA. The extremely high expression of miR-21 was confirmed for both mock and infected samples; miR-21 was expressed at about 50 fold higher level than U6 RNA and between 3,000 and 20,000 fold higher than porcine miRNAs (Figure S2).

### Differential expression of porcine small RNAs between PRV-infected and mock-infected DCs

MiRNAs as well as other small regulatory molecules were found to be differentially expressed between PRV-infected and mock-infected DCs. A statistical test based on a moderate negative binomial dispersion was applied, and after correction for False Discovery Rate (FDR, p<0.05), a total of 24 porcine sequence reads were identified as differentially expressed between the two conditions. Considering a more stringent FDR threshold (p<0.01) 15 differentially expressed sequence tags remained. The differentially expressed reads were mapped against the non-redundant set of nucleotide sequences at NCBI (http://www.ncbi.nlm.nih.gov/nucleotide/) and miRBase.

Small molecules up-regulated in infected samples were only barely detected in mock-infected samples (Table 4). The most up-regulated one (35 folds more) was snoRNA Z40, an orthologue of human mgh28S-2411 (or SORD6) predicted to guide 2′O-ribose methylation of 28S rRNA Gm2411 [34]. Other small molecules were more slightly over-expressed (between 1.8 and 4.0 folds) and included another snoRNA of the box C/D class (D81, homologous to human U81) involved as well in guiding 20-O-ribose methylation of 28S rRNA [34], four miRNAs (miR-192, miR-194, miR-215, and let-7b) and four mitochondrial tags. PRV infection was also associated to intense down-regulation (up to 31 folds) of tags mapping to the 28S ribosomal rRNA as well as of four miRNAs (miR-27b*, miR-29a, miR-29b-1* and miR-30e). All these tags were expressed at high level in the physiological non infected condition (Table 4).

**Table 4:** Differential expression analysis of porcine small RNAs.

	*Non infected samples*			*Infected samples*				
Annotation	GDI3	GDI5	GDI7	GDI4	GDI6	GDI8	Fold change	Adj.p
Z40 snoRNA	1	0	0	52	41	120	35.33	7.43E-13
ssc-miR-192	0	0	1	2	7	16	4.00	2.82E-05
mitochondrion	0	0	0	9	13	1	3.83	8.45E-06
mitochondrion	0	0	0	7	3	4	2.33	3.30E-03
mitochondrion	0	0	0	1	9	1	1.83	3.78E-02
mitochondrion	0	4	0	9	4	7	2.67	4.28E-02
hsa-miR-194	0	1	2	10	1	15	3.83	7.00E-04
ssc-miR-215	0	6	3	3	10	15	3.17	3.06E-02
hsa-let-7b	0	4	8	14	7	10	3.17	1.86E-02
D81 snoRNA	0	1	1	5	8	10	3.50	5.00E-04
ssc-miR-27b*	63	74	42	10	3	3	-27.17	4.90E-24
hsa-miR-29b-1*	8	18	31	2	3	1	-8.50	6.91E-06
28S ribosomal RNA	122	87	134	51	59	47	-31.00	7.82E-05
ssc-miR-29a	35	86	87	28	31	32	-19.50	5.20E-03
ssc-miR-30e-3p	53	55	31	24	13	24	-13.00	3.92E-02

Sequence count of reads identified as differentially expressed with edgeR method. The first column contains the current annotation of small RNAs based on sequence alignment to the non redundant nucleotide collection at NCBI. The last column contains the adjusted p-values (adj. p value) of differential expression significance.

### Biological networks and functions affected by differentially expressed porcine miRNAs

Differentially expressed swine miRNAs were analysed to determine which gene networks and functions are possibly regulated by these miRNAs. Among the eight porcine miRNA that were found differentially expressed, seven (all but miR-192, for which no interacting molecule in Ingenuity Pathway Knowledge Base was found) were eligible for network analysis with the Ingenuity Pathway Analysis tool (Ingenuity® Systems, www.ingenuity.com).

**Figure 2:**
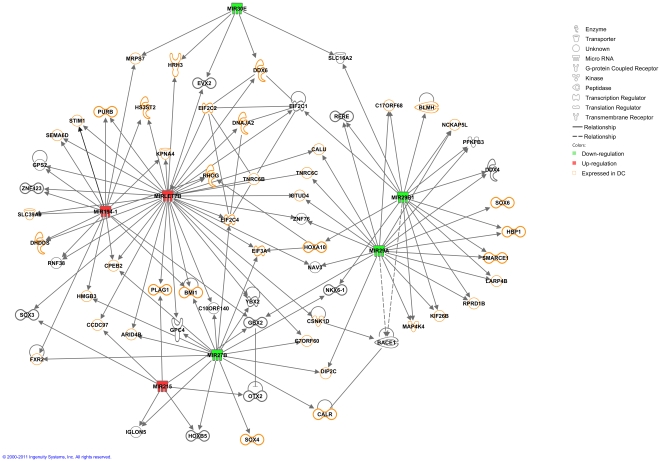
Significant gene network of porcine miRNA predicted targets. Putative gene targets and relationships defined by porcine miRNAs differentially expressed in infected and mock-infected DCs. For miRNAs over-expressed in mock-infected samples molecule symbols are coloured in green and for miRNAs over-expressed in infected samples molecule symbols are coloured in red. For genes known to be expressed in DCs, the molecule symbols are represented with yellow borders. Relationships between miRNAs and genes are defined based on IPKB.

A unique highly significant network (score: 17) containing the seven eligible miRNAs was obtained (Figure 2). Based on expression data available in *Ingenuity Pathway Knowledge Base* (IPKB), 41 genes were found to be expressed in DCs among a total of 70 genes included in the network. Most genes belonging to the network were directly connected to at least two miRNAs. Five miRNAs were nodal and were either up-regulated (miR-194 and miR-let7b) or down-regulated (miR-27B, miR29-a and miR29-b1).

Several functions associated with this network were significant (Table S3). The first most significant functions were nervous system development and function (p-value = 1.6E-05 – 2.92E-02), followed by organ development (p-value = 1.6E-05 – 2.72E-02) and infection mechanism (3.13E-04). Among the genes involved in nervous system development and function and organ development, BMI1 (Polycomb ring finger oncogene), GBX2 (Gastrulation brain homeobox 2), OTX2 (Orthodenticle homeobox 2), SOX4 (SRY-box 4), ZNF423 (Zinc finger protein 423) and NKX6-1 (NK6 homeobox 1) participate to the development of nervous organs or cells, migration of neurons or neurogenesis. Three genes (EIF2C1, EIF2C2 and EIF2C4) encoding subunits of the eukaryotic translation initiation factor 2C have been previously reported to play a role in infection mechanism (Table S3). These three molecules were directly connected to miR-let7b, that was up-regulated. EIF2C1 and EIF2C4 were respectively connected to miR29b and miR-27b, both down-regulated.

The IPA network was overlaid with the data previously obtained on differential gene expression between PK15 cells infected by PRV (8h post-infection) and mock-infected cells [16] (Figure S3). Thirteen and eight genes from the network were up- and down-regulated in PK15 cells infected by PRV, respectively. Interestingly, among the thirteen up-regulated genes, eleven are reported in IPKB to be expressed in DCs and, among the eight down-regulated genes, six are shown to be expressed in DCs. The down-regulated miR-29b and miR-29a are both or each connected to up-regulated SMARCE1, HBO1, NKX6-1, HOXA10, HBP1, OTUD4, that could be further considered as potent targets during infection. Moreover, EIF2C1 and EIF2C2 have been found over and under-expressed in infected PK15, respectively (Figure S3).

## Discussion

The deep sequencing approach adopted in this study has been very effective for profiling the miRNA transcriptome of porcine DCs infected by the PRV, with over 96% reads aligned with a high mapping quality to the genome of the pig or the virus. This is in line with the increasing number of reports which have successfully applied deep sequencing for profiling miRNAs in other biological systems e.g. [35], [36].

The comparative and biogenesis criteria used to analyse the deep sequencing data contributed to predict several new swine miRNAs. Besides the subset of known swine miRNAs found to be modulated in porcine DCs (156 out of a total of 211 annotated in miRBase v.16), the comparative analysis identified more than three hundred miRNAs with high similarity to annotated miRNAs in other mammalian species (Table S2). For example, only six members of Let-7 miRNA family are currently annotated for the pig (miRBase, release 16), however by comparative analysis of porcine sequence tags with human miRNAs all the eleven members of this miRNA family could be identified by the present study. In addition the deep sequencing approach facilitated the prediction of 27 new putative porcine miRNAs which lacked conservation with other mammals and may be specific of the suid lineage (Table S3). Overall, these findings confirmed that the current annotation of porcine miRNAs is still incomplete and that many more pig specific miRNAs are likely to exist even though not yet characterized.

The analysis of the deep sequencing data revealed that miR-21 expression was very high, both in infected and mock infected cells at 8 h PI, compared to other miRNAs, and this finding was confirmed by qPCR tests on independent samples. MicroRNAs are known to be differentially expressed depending on the developmental stage and tissue [37] and the high expression of miR-21 may be related to cell differentiation. The DCs analyzed in this study were obtained by *in vitro* differentiation of monocytes purified from peripheral blood mononuclear cells (PBMCs), and miR-21 has been previously reported as being involved in phenotypic and functional differentiation of Monocyte-Derived Dendritic Cells (MDDC) [38], [39]. Indeed, other small RNA deep sequencing studies have shown a high predominance of single miRNAs in different systems (such as. let-7, miR-21 and others) [40], [41].

The analyses brought to the prediction of five new viral miRNAs mapping to the latency region of PRV and to a preliminary identification of their putative viral gene targets (Figure 1, Table 2, Table 3). In other herpesviruses, the miRNAs expressed by the Large Latency Transcript (LAT) down-regulate the expression of key viral immediate-early or early regulatory proteins [42] and it has been hypothesized that LAT-encoded miRNAs function as a molecular switch between HSV latency and reactivation in infected human ganglia [4], [43]. In particular in HSV-2, the LAT-encoded miRNAs in latently infected cells down-regulate the immediate-early transactivators ICP0 and ICP34.5 [44]. ICP0 functions as potent activator of HSV-1 and HSV-2 lytic replication [2], [45] while ICP4 has been observed to be down-regulated by LAT transcripts and to be involved in latency maintenance in HSV-1 [46]. Given that the EP0 and PRV IE180 putative target genes of PRV are respectively homologous to ICP0 and ICP4 in HSV [47], [18] the miRNAs expressed from the LLT of PRV may repress both proteins, facilitating the establishment and/or maintenance of the latent state in PRV.

The miRNAs expressed by the LLT of PRV were the most abundant viral sequence reads in DCs productively infected by PRV, i.e. releasing virions at 8 h pi. As this system does not mimic the PRV latency stage, this might mean that at least the first steps of the preparation of the virus to the latency stage occurs early during the lytic phase and *in vitro*. It has been reported that HSV-1 can establish latency only in primary neurons, an environment that has so far proven impossible to fully recapitulate in culture, however the LAT-encoded miRNAs of HSV-1 are also expressed in the HeLa and Vero cells cell lines during productive replication [45]. In PRV, it has been reported that transcripts of different length are transcribed by the LLT region during the lytic infection of cultured mammalian (PK15 and MDBK) cells [45].

The differentially expressed cellular miRNAs provided additional clues on latency initiation in DCs. It is striking that a unique network of predicted gene targets was drawn (Figure 2). More than half of these molecules have been shown to be expressed in DCs, strongly suggesting that the network is specific of gene expression in DCs as well as of PRV infection. Due to the complexity of interactions between miRNAs and their common targets it is difficult to infer a clear link between up/down-regulated miRNAs and up/down-regulated genes (Figure 2), although it should be noted that infection caused an intense down-regulation of miRNAs which were well expressed in the mock infected condition (Table 4).

The six most significant target genes connected to the differentially expressed swine miRNAs (BMI1, GBX2, OTX2, SOX4, ZNF423, NKX6-1) play a role in nervous system development and function (Figure 2). Since the primary sites of PRV latency are the sensory neurons of the trigeminal ganglia [15] the miRNAs-mediated modulation of these genes strongly indicate that at least the first steps of latency are activated in dendritic cells (Table S4). Among these genes, BMI1 encodes a member of the Polycomb repressive complex 1 protein which is involved in DNA methylation and is able to bind to the herpes simplex virus 1 latent genome [48]. Furthermore, all the six genes have roles in cell growth, proliferation and death, e.g. BMI1 [49]. It has been reported that PRV infection modulates the genes of the apoptosis pathway belonging to the BCL-2 family in PK15 cells [42] and it has been suggested that the anti-apoptotic transcription factor Brn-3a may be of importance in the survival of neurons of the trigeminal ganglia during PRV infection [42]. Indeed, many herpesviruses have the ability to block apoptosis during the latent and reactivation phases of infection [50] and it has been recently shown that several apoptosis-associated genes were decreased in expression during latent infection of chicken splenocytes by the Marek's disease virus [51].

Other three genes of the network (EIF2C1, EIF2C2 and EIF2C4) were modulated by PRV infection. These genes encode Argonaute 1, 2 and 4 proteins, which participate to the RNA-induced silencing complex (RISC) and tightly bind the single strand miRNA in the cytoplasm before their binding to the cellular or viral target mRNA [4]. The RNAi-miRNA machinery itself seems indeed under miRNA control. In particular, human EIF2C1 mRNA is a predicted target of two human miRNAs (miR-let7b and miR29b) and EIF2C4 mRNA is a predicted target of human miR-let7b [29]. It has been reported that E2 envelope protein of the hepatitis C virus is able to interact with Argonaute 2 and to suppress RNA interference [52].

Overall, these results provide evidence that both the host and viral miRNA regulation layers are modulated upon PRV infection of the PRV-DCs experimental system, and strongly suggest that their interplay is part of the process to establish latency. This is supported by the acquired knowledge in several other herpesviruses and contribute to the resuming of microRNAs functional roles and their interplay in the host-pathogen system.

## Materials and Methods

### Experimental system

The virulent wild type NIA3 strain of PRV was grown by infecting confluent monolayers of the porcine PK15 epithelial cell line as previously described [53]. Porcine immature DCs were differentiated *in vitro* from blood monocytes isolated from PBMCs by immune-magnetic sorting using the Anti-CD172 monoclonal antibody and treated during 3–4 days with a mixture of recombinant porcine granulocyte-macrophage colony stimulating factor and interleukin 4 [NCP and FL, [23]. Three batches of DCs produced from 3 Large White pigs were either mock-infected or infected with PRV NIA3 at a MOI of 3. For each infection experiment, total RNA samples were obtained from infected and mock-infected cells at 8 hours post-infection and checked for quantity and quality (Nanodrop, Agilent).

### Sources of sequences and genome sequence assemblies

The viral strain used for infection and subsequent analysis is the NIA-3, a virulent strain of the PRV families. PRV full genome (NCBI Reference Sequence: NC_006151.1), is a composite sequence of six strains and is 143461 bp long. *Sus scrofa* draft genome assembly (Sscrofa8) has been produced by the International Swine Genome Sequencing Consortium and was retrieved from www.ensembl.org. *Sus scrofa*, full genome, chromosome 1-18 and X, total size: 1884975130 bp.

### Deep sequencing and data analysis

RNA samples were processed *in vitro* to generate libraries of short inserts, using the DNA Colonies Template Library system. The libraries were prepared using “bar-codes” to track their origin, and to facilitate the analysis of 6 samples (2×3 biological replicates) in two Genome Analyzer (GA) channels. Quality control was performed and the pooled libraries were then sequenced on the Illumina Genome Analyzer GAII generating millions of 32 bases reads (FASTERIS SA, Plan-les-Ouates, Switzerland).

After 36 sequencing cycles, the sequence reads were analysed and reads from different samples separated according to their bar code, and the adapter sequences were trimmed. The MAQ program [24] was used to map the inserts of 21 to 24 bases (with a mean of 1.8 million sequences per sample) with a maximum set at 2 mismatches on the pig and viral reference sequences. Inserts mapping to several positions on the references with the same “mapping quality” (i.e. number of mismatches and quality of the bases generating the mismatches) were attributed at random to one of them and attributed a “0” mapping quality.

### MicroRNA discovery

Known microRNAs in the sequence data set were identified using miRBase as reference database (release 16; http://www.mirbase.org/).

To define a small RNA sequence as a microRNA both expression and biogenesis criteria were evaluated, based on the assumption that the presence of a small RNA sequence is in itself proof of expression. The biogenesis criterion was evaluated using the software miRDeep which is specifically designed for the analysis deep sequencing data [26] and which allows the prediction of pri- and pre- mRNA secondary structure stability through the RNAfold algorithm [54]. The algorithm uses a probabilistic model of miRNA biogenesis to score compatibility of the position and frequency of sequenced RNA with the secondary structure of the microRNA precursor. miRDeep maps the sequenced reads to the genome and to the corresponding predicted miRNA precursor hairpin structure. Only reads that are compatible with the Dicer processing are retained [26].

### Gene target analysis

The miRanda algorithm was used to find genomic targets for microRNAs. This algorithm, written in C, is available as open-source [40]. Code can be downloaded from http://cbio.mskcc.org/research/sander/data/miRNA2003/miranda_new.html. The algorithm performs a dynamic local alignment between the query miRNA sequence and the reference sequence where G:U wobble is allowed and generates scores for the alignments found. Parameters used in the command line to filter most significant results were: -sc 130 -en 30.

The target genes predicted by miRanda were verified by TargetScan and PITA software. TargetScan [30] is specifically designed to identify vertebrate microRNAs, but it can be used to predict conserved and non conserved miRNA targets using a custom set of data. Targets are identified based on their match to positions 2-8 of the mature miRNA (m8: the seed + position 8). Perl codes were downloaded from http://www.targetscan.org/cgi-bin/targetscan/data_download.cgi?db=vert_50.

PITA (Probability of Interaction by Target Accessibility) [31] identifies putative targets assigning an energetic score (ΔΔG). Only sites with ΔΔG values below −10 were considered as significant. PITA executable can be downloaded from http://genie.weizmann.ac.il/pubs/mir07/mir07_exe.html.

### Differential expression analysis

To assess the statistical significance of small RNAs differentially expressed in infected and mock - infected cells all the PRV viral sequences which would have been identified as differentially expressed were excluded. Sequence tags which showed expression in all biological replicates for infected or non infected cells were considered. In total of 3211 sequence tags were further analyzed.

Statistical analyses were performed with custom scripts written in R software (EdgeR http://bioinf.wehi.edu.au/resources/ [55]). A statistical test was applied to evaluate differential expression of data deriving from Digital gene expression (DGE) data, in particular where a limited number of replicates are available. The method was based on the negative binomial distribution, and uses conditional weighted likelihood to moderate the level of overdispersion across sequence tags.

### Functional and network analyses

The *Ingenuity Pathway Analysis* (IPA) software v7.0 (Ingenuity Systems Inc., USA, http://www.ingenuity.com/) was used for the functional and network analyses of the eight differentially transcribed porcine miRNA. The network analysis aimed at searching for interactions (known from the literature) between differentiated genes and all other molecules (genes, gene products or small molecules) contained in *Ingenuity Pathway Knowledge Base* (IPKB). microRNA-mRNA targeting interactions are retrieved from ARGONAUTE 2 (miRWalk: http://www.ma.uni-heidelberg.de/apps/zmf/mirwalk/) and TarBase (http://diana.cslab.ece.ntua.gr/tarbase/) databases. For a given network (containing at most 70 molecules in our analysis), the degree of association is estimated by considering the proportion of eligible genes (genes with at least one interaction with another full length wild type gene or protein in IPKB). From the right-tailed Fisher exact test performed with IPA, only those networks with a score (log(1/P-value)) greater than 3 were considered as significant to the differentiated genes. For significant networks, the top functions and diseases are reported. In addition, differential expression levels of PK15 cells infected by PRV compared to mock-infected cells [16] and significant network were overlaid and genes expressed in DC were highlighted using IPKB expression data.

### Quantitative real time PCR (qPCR)

In order to test the level of expression of miR21 vs. other host miRNAs, RNA samples were obtained from infected and mock-infected DCs at 0, 4 and 12 hours post-infection challenged under the same experimental conditions (see ‘Experimental system’). MicroRNA expression was tested with the Mercury LNA microRNA PCR system (Exiqon, USA). Triplicate reactions were set up in a 10 µl final volume containing 10 ng of total RNA as template and the set of commercial primers and probes for miR-21, miR-339-3p, miR-184, miR-7, miR-370, miR-708*, miR-29b-1* and U6 small nuclear RNA (Exiqon, USA). Reactions were run on a 7900 HT FAST real time PCR system (Applied Biosystems, USA).

## Supporting Information

Figure S1 **Hairpin structures of the predicted viral pre-miRNAs.** Hairpin structures of the viral miRNAs were predicted with the RNAfold software. The associated minimum free energy (MFE) value is reported. Both dot-bracket and graphical notation are displayed for each pre-miRNA.
(DOC)

Figure S2 **Expression level of miR-21 compared to other porcine miRNAs and to U6 small nuclear RNA.** The graph displays the qPCR mean Ct values of miR-21 in porcine DCs compared to those of other six porcine miRNAs (miR-339-3p, miR-184, miR-7, miR-370, miR-708 and miR-29b-1*) and of the U6 small RNA molecule. Reactions were carried out using equal amounts of total template RNA obtained from DC samples challenged with PRV (4 h and 12 h PI) vs. uninfected control DCs (0 h PI). The low Ct values obtained for miR-21 expression confirmed that this miRNA was expressed several folds more than other miRNAs and U6 small RNA molecule in both infected and uninfected samples. The high Ct values obtained for the other miRNAs reflect their very low level of expression.
(DOC)

Figure S3 **Significant gene network overlaid with DCs gene expression and with differential expression levels between PRV infected and mock-infected PK15 cells 8 h PI.** Putative gene targets and relationships defined by porcine miRNAs differentially expressed in infected and mock-infected dendritic cells. Genes expressed in DCs appear in orange according to the expression data contained in *Ingenuity Pathways Knowledge Base*. The data on expression levels of genes differentially expressed between PRV infected and mock-infected PK15 epithelial cells previously obtained (Flori et al, 2008, BMC Genomics) were used to input information on the expression of miRNA target genes.
(TIF)

Table S1 **Swine miRNAs expressed in DCs.** Swine miRNAs expressed in each sequenced sample of DCs (mock-infected: GDI3. GDI5. GDI7; infected by PRV: GDI4. GDI6. GDI8). For each miRNA, the number of reads mapping to the pig genome and the mean frequency are reported. Swine miRNAs were identified using miRBase as reference database (release 16; http://www.mirbase.org/). The most abundant identified miRNA was miR-21, followed by two members of the let-7 family (ssc-let-7f, ssc-let-7c) known to be the most abundantly expressed in higher eukaryotes.
(DOC)

Table S2 **Orthologous miRNAs.** List of orthologous miRNAs identified through alignment with mature and pre- miRNAs (considered organisms: *Homo sapiens, Mus musculus, Bos taurus, Canis familiaris, Equus caballus* and *Gallus gallus*). MiRNA sequences were retrieved from miRBase, release 16 (ftp://mirbase.org/pub/mirbase/CURRENT/).
(XLS)

Table S3 **Newly predicted swine miRNAs in DCs.** Mature sequence and sequence read count of new porcine miRNAs predicted with miRDeep in each sequenced sample of DCs (mock-infected: GDI3, GDI5, GDI7; infected by PRV: GDI4, GDI6, GDI8). In the first part of the table miRNA 21–24 nt long are listed, while the second part shows miRNA of less common length (<21 or >24).
(DOC)

Table S4 **List of the significant functional annotations of genes belonging to the significant IPA network (p-value <0.05).**
(XLS)
