# Supplementary material for: Co-Expression of Host and Viral MicroRNAs in Porcine Dendritic Cells Infected by the Pseudorabies Virus
Source: PLoS One. 2011 Mar 8;6(3):e17374. doi: 10.1371/journal.pone.0017374 (PMC3050891; doi:10.1371/journal.pone.0017374)
Supplement: Figure S1 — Hairpin structures of the predicted viral pre-miRNAs. Hairpin structures of the viral miRNAs were predicted with the RNAfold software. The associated minimum free energy (MFE) value is reported. Both dot-bracket and graphical notation are displayed for each pre-miRNA. (DOC) [file pone.0017374.s001.doc]

**Supporting Figure 1. Pre-miR hairpin structures.**

Method: RNAfold webserver [1]

**prv-miR-1**

mature: TCTCACCCCTGGGTCCGTCGC

pre-miR-1:GACGGCTCCTGGGTCTGAAAGCGGCGCTGCGGATCCCCCGCTCTCACCCCTGGGTCCGTCGC

**RNA-fold**

GACGGCUCCUGGGUCUGAAAGCGGCGCUGCGGAUCCCCCGCUCUCACCCCUGGGUCCGUCGC

(((((..((..((..(((.(((((.............))))).)))..))..)).)))))..

minimum free energy: -28.82 kcal/mol


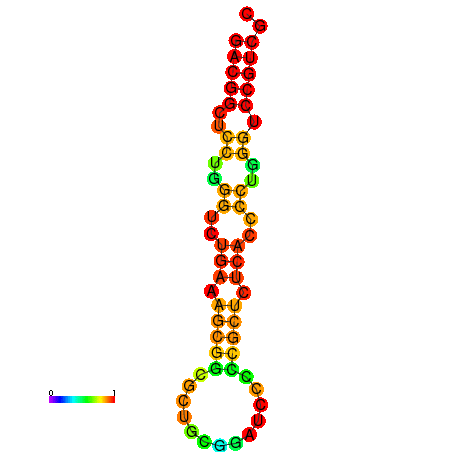


**Base-pair probabilities**

**prv-miR-2**

mature: CGTACCGACCCGCCTACCAGGCA

pre-miR-2: CGTACCGACCCGCCTACCAGGCAGGCTAGAAGCCTCACGCTGCTGCTTGGCAGCGGGTGGGTACCCA

**RNA-fold**

CGUACCGACCCGCCUACCAGGCAGGCUAGAAGCCUCACGCUGCUGCUUGGCAGCGGGUGGGUACCCA

.(((((.(((((((.....)))((((.....))))...((((((....)))))))))).)))))...

minimum free energy: -36.70 kcal/mol


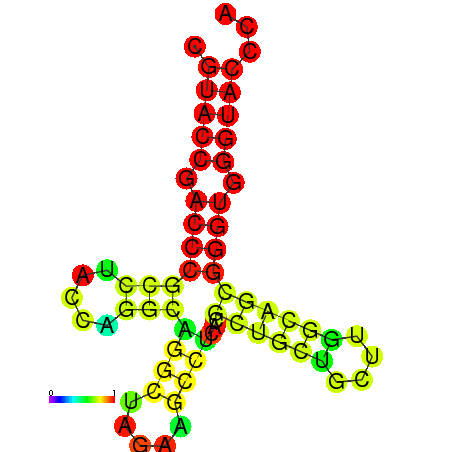


Base-pair probabilities

**prv-miR-3**

mature: ATGAGTGGATGGATGGAGGCGA

pre-miR-3: GTGTCCCCTGTTCCCCATCCTTCCACCCATACATTCATCCGTGCGGTGGTGGGGATGAGTGGATGGATGGAGGCGA

**RNA-fold**

GUGUCCCCUGUUCCCCAUCCUUCCACCCAUACAUUCAUCCGUGCGGUGGUGGGGAUGAGUGGAUGGAUGGAGGCGA

.................((((((((.((((.(((((((((.(((....))).))))))))).)))).)))))).))

minimum free energy: -31.10 kcal/mol


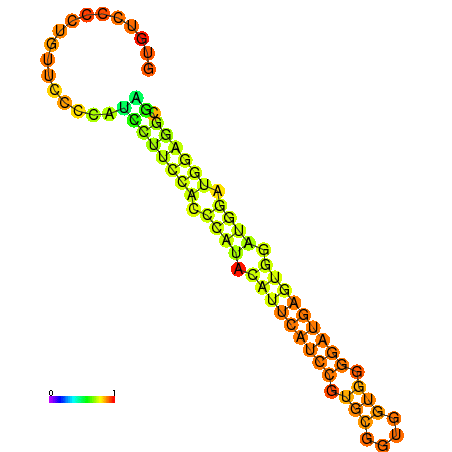


**Base-pair probabilities**

**prv-miR-4**

mature: CGGAACCGGGTGCAGGCG

pre-miR-4:

CGGAACCGGGTGCAGGCGGTGGGGGCGAAGATTGGGTTGGGTGAGAGACTAGAACCGGTGTTCTCAACCCTTCTGGAGCCCTACCCTCTGTGCCTGGACTTTCCAGC

**RNA-fold**

CGGAACCGGGUGCAGGCGGUGGGGGCGAAGAUUGGGUUGGGUGAGAGACUAGAACCGGUGUUCUCAACCCUUCUGGAGCCCUACCCUCUGUGCCUGGACUUUCCAGC

.(((((((((..((((.((((((.((..(((..((((((((...((.(((......))).)))))))))).)))...)))))))).))))..)))))...))))...

minimum free energy: -53.60 kcal/mol

**
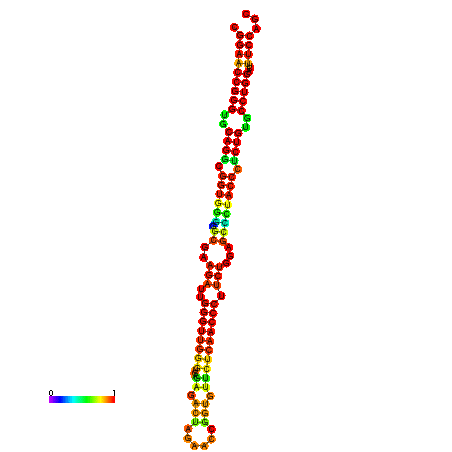
**

**Base-pair probabilities**

**prv-miR-5**

mature: ATCGAGGAGATGTGGAGGGGTGC

pre-miR-5: ATCGAGGAGATGTGGAGGGGTGCCAAGCGCCCGCCGCCCTCCCCCGCATCTCTTCTCTCT

**RNA-fold**

AUCGAGGAGAUGUGGAGGGGUGCCAAGCGCCCGCCGCCCUCCCCCGCAUCUCUUCUCUCU

...((((((((((((.((((.((...((....)).)).)))).)))))))))))).....

minimum free energy: **-30.50** kcal/mol


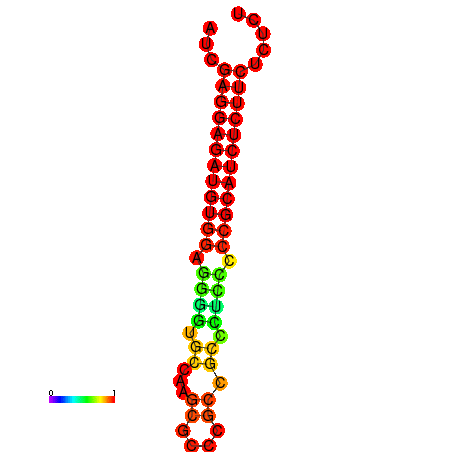


**Base-pair probabilities**

**Reference:**

1. Gruber AR, Lorenz R, Bernhart SH, Neubock R, Hofacker IL (2008) The Vienna RNA websuite. Nucleic Acids Res 36: W70-74.
