# Supplementary material for: Co-Expression of Host and Viral MicroRNAs in Porcine Dendritic Cells Infected by the Pseudorabies Virus
Source: PLoS One. 2011 Mar 8;6(3):e17374. doi: 10.1371/journal.pone.0017374 (PMC3050891; doi:10.1371/journal.pone.0017374)
Supplement: Figure S2 — Expression level of miR-21 compared to other porcine miRNAs and to U6 small nuclear RNA. The graph displays the qPCR mean Ct values of miR-21 in porcine DCs compared to those of other six porcine miRNAs (miR-339-3p, miR-184, miR-7, miR-370, miR-708 and miR-29b-1*) and of the U6 small RNA molecule. Reactions were carried out using equal amounts of total template RNA obtained from DC samples challenged with PRV (4 h and 12 h PI) vs. uninfected control DCs (0 h PI). The low Ct values obtained for miR-21 expression confirmed that this miRNA was expressed several folds more than other miRNAs and U6 small RNA molecule in both infected and uninfected samples. The high Ct values obtained for the other miRNAs reflect their very low level of expression. (DOC) [file pone.0017374.s002.doc]

### Supporting Figure 2. Expression level of miR-21 compared to other porcine miRNAs and to U6 small nuclear RNA.
