# Supplementary figures and images for: Co-Expression of Host and Viral MicroRNAs in Porcine Dendritic Cells Infected by the Pseudorabies Virus
Source: PLoS One. 2011 Mar 8;6(3):e17374. doi: 10.1371/journal.pone.0017374 (PMC3050891; doi:10.1371/journal.pone.0017374)

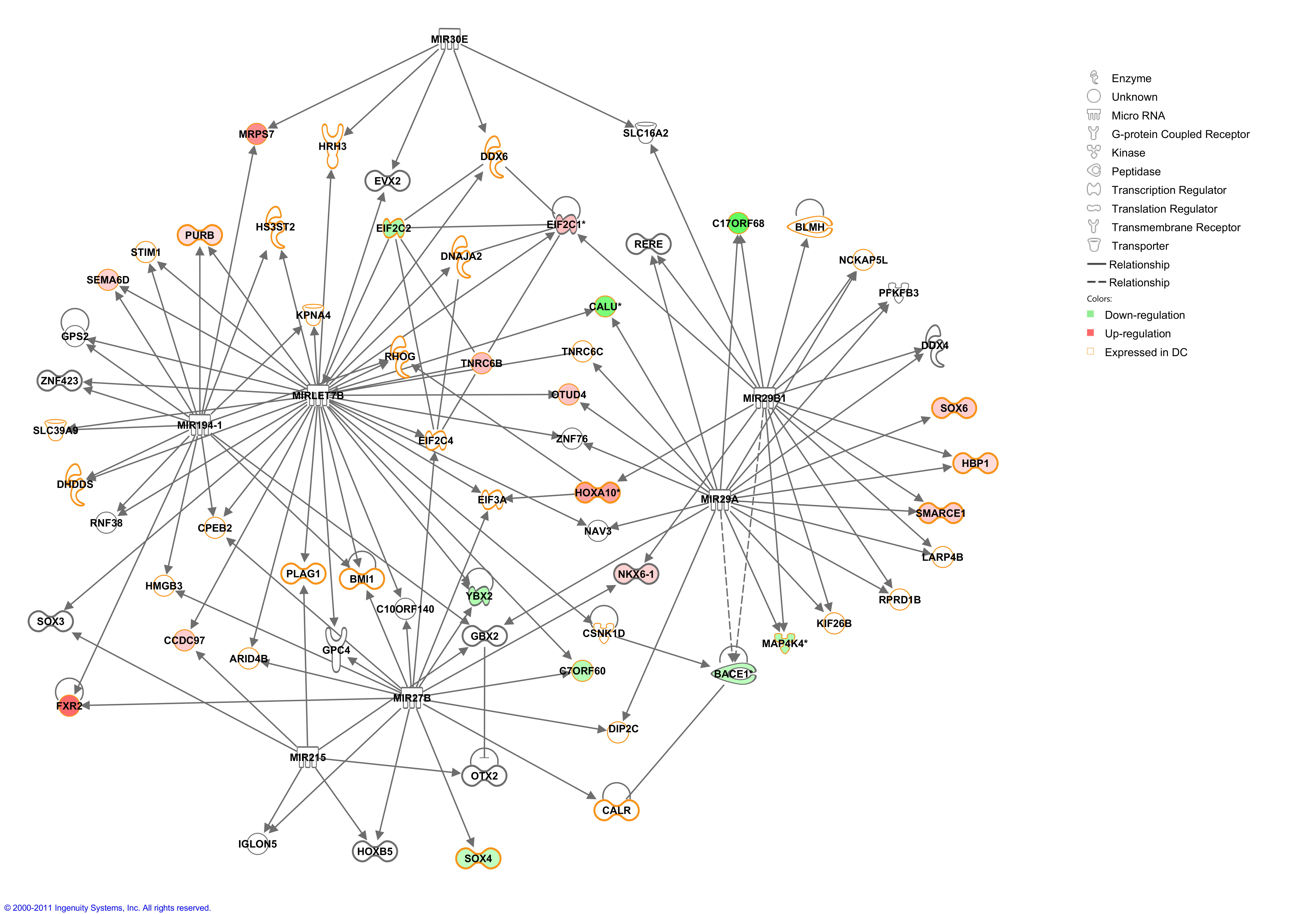

Supplement: Figure S3 — Significant gene network overlaid with DCs gene expression and with differential expression levels between PRV infected and mock-infected PK15 cells 8 h PI. Putative gene targets and relationships defined by porcine miRNAs differentially expressed in infected and mock-infected dendritic cells. Genes expressed in DCs appear in orange according to the expression data contained in Ingenuity Pathways Knowledge Base. The data on expression levels of genes differentially expressed between PRV infected and mock-infected PK15 epithelial cells previously obtained (Flori et al, 2008, BMC Genomics) were used to input information on the expression of miRNA target genes. (TIF) [file pone.0017374.s003.tif]
