# Supplementary material for: Co-Expression of Host and Viral MicroRNAs in Porcine Dendritic Cells Infected by the Pseudorabies Virus
Source: PLoS One. 2011 Mar 8;6(3):e17374. doi: 10.1371/journal.pone.0017374 (PMC3050891; doi:10.1371/journal.pone.0017374)
Supplement: Table S1 — Swine miRNAs expressed in DCs. Swine miRNAs expressed in each sequenced sample of DCs (mock-infected: GDI3. GDI5. GDI7; infected by PRV: GDI4. GDI6. GDI8). For each miRNA, the number of reads mapping to the pig genome and the mean frequency are reported. Swine miRNAs were identified using miRBase as reference database (release 16; http://www.mirbase.org/). The most abundant identified miRNA was miR-21, followed by two members of the let-7 family (ssc-let-7f, ssc-let-7c) known to be the most abundantly expressed in higher eukaryotes. (DOC) [file pone.0017374.s004.doc]

### Supporting Table 1. Swine miRNAs expressed in DCs

Swine miRNAs expressed in each sequenced sample of DCs (mock-infected: GDI3. GDI5. GDI7; infected by PRV: GDI4. GDI6. GDI8). For each miRNA, the number of reads mapping to the pig genome and the mean frequency are reported. Swine miRNAs were identified using miRBase as reference database (release 16; <http://www.mirbase.org/>). The most abundant identified miRNA was miR-21, followed by two members of the let-7 family (ssc-let-7f, ssc-let-7c) known to be the most abundantly expressed in higher eukaryotes.

|  | **Animal 1** | | **Animal 2** | | **Animal 3** | |  |
| --- | --- | --- | --- | --- | --- | --- | --- |
| **ssc-miR** | **GDI-3** | **GDI-4** | **GDI-5** | **GDI-6** | **GDI-7** | **GDI-8** | **mean frequency** |
| ssc-miR-21 | 1575806 | 1282670 | 1899707 | 1256212 | 1942270 | 1508436 | 0,95530 |
| ssc-let-7f | 29233 | 19842 | 29897 | 50166 | 28718 | 21355 | 0,01809 |
| ssc-let-7c | 9893 | 5225 | 6531 | 11120 | 5356 | 3997 | 0,00425 |
| ssc-miR-27b | 4542 | 2500 | 1504 | 2577 | 2675 | 1100 | 0,00150 |
| ssc-let-7a | 3967 | 2205 | 3602 | 5883 | 2652 | 2191 | 0,00207 |
| ssc-let-7g | 3668 | 2498 | 3237 | 6587 | 3914 | 2430 | 0,00225 |
| ssc-miR-23a | 2557 | 4544 | 1134 | 3412 | 5369 | 1016 | 0,00182 |
| ssc-miR-23b | 1463 | 2751 | 1661 | 1792 | 2869 | 1203 | 0,00118 |
| ssc-miR-98 | 1303 | 651 | 713 | 1973 | 1062 | 686 | 0,00064 |
| ssc-miR-27b* | 979 | 219 | 556 | 452 | 552 | 193 | 0,00030 |
| ssc-miR-30d | 859 | 527 | 630 | 634 | 783 | 439 | 0,00039 |
| ssc-miR-26a | 835 | 1151 | 582 | 845 | 1590 | 409 | 0,00055 |
| ssc-miR-191 | 811 | 681 | 787 | 751 | 955 | 614 | 0,00046 |
| ssc-miR-362 | 699 | 924 | 565 | 474 | 1442 | 532 | 0,00047 |
| ssc-miR-27a | 674 | 142 | 40 | 587 | 193 | 39 | 0,00017 |
| ssc-miR-148a | 629 | 1488 | 1495 | 1372 | 2848 | 1199 | 0,00091 |
| ssc-miR-222 | 593 | 1686 | 3933 | 3784 | 6295 | 4480 | 0,00210 |
| ssc-miR-320 | 554 | 2645 | 1200 | 1226 | 6496 | 991 | 0,00132 |
| ssc-miR-24 | 534 | 888 | 898 | 415 | 1288 | 721 | 0,00048 |
| ssc-miR-378 | 409 | 608 | 1430 | 618 | 871 | 792 | 0,00048 |
| ssc-miR-15b | 361 | 487 | 359 | 329 | 638 | 289 | 0,00025 |
| ssc-miR-221 | 318 | 344 | 1269 | 619 | 1238 | 1336 | 0,00052 |
| ssc-miR-29c | 277 | 363 | 443 | 296 | 482 | 305 | 0,00022 |
| ssc-miR-29a | 227 | 366 | 928 | 415 | 1021 | 518 | 0,00035 |
| ssc-miR-374a | 223 | 2077 | 1497 | 294 | 2890 | 936 | 0,00080 |
| ssc-miR-30b-5p | 217 | 254 | 179 | 272 | 393 | 103 | 0,00014 |
| ssc-miR-19a | 197 | 205 | 170 | 222 | 350 | 89 | 0,00012 |
| ssc-miR-425-5p | 195 | 212 | 341 | 129 | 266 | 251 | 0,00014 |
| ssc-miR-1306-3p | 142 | 148 | 125 | 96 | 220 | 91 | 0,00008 |
| ssc-miR-186 | 141 | 579 | 423 | 177 | 1005 | 338 | 0,00027 |
| ssc-miR-140* | 126 | 257 | 378 | 204 | 351 | 237 | 0,00016 |
| ssc-miR-20 | 124 | 10 | 13 | 165 | 23 | 2 | 0,00003 |
| ssc-miR-146b | 122 | 224 | 148 | 72 | 528 | 180 | 0,00013 |
| ssc-miR-15a | 118 | 79 | 77 | 149 | 111 | 45 | 0,00006 |
| ssc-miR-30e-5p | 116 | 92 | 114 | 89 | 120 | 93 | 0,00006 |
| ssc-miR-29b | 114 | 161 | 138 | 274 | 448 | 138 | 0,00013 |
| ssc-miR-363 | 112 | 101 | 169 | 130 | 138 | 115 | 0,00008 |
| ssc-let-7e | 108 | 82 | 122 | 252 | 211 | 144 | 0,00009 |
| ssc-miR-125b | 105 | 163 | 265 | 149 | 340 | 176 | 0,00012 |
| ssc-miR-142 | 103 | 590 | 483 | 159 | 1278 | 429 | 0,00031 |
| ssc-miR-30e-3p | 93 | 71 | 115 | 37 | 83 | 61 | 0,00005 |
| ssc-miR-374b-5p | 90 | 254 | 213 | 82 | 398 | 132 | 0,00012 |
| ssc-miR-18 | 90 | 36 | 19 | 167 | 66 | 15 | 0,00004 |
| ssc-miR-103 | 76 | 266 | 312 | 101 | 474 | 354 | 0,00016 |
| ssc-miR-340 | 66 | 78 | 66 | 54 | 94 | 26 | 0,00004 |
| ssc-miR-1 | 54 | 50 | 127 | 32 | 88 | 99 | 0,00005 |
| ssc-miR-122 | 48 | 5 | 16 | 116 | 17 | 11 | 0,00002 |
| ssc-miR-301 | 47 | 11 | 17 | 43 | 14 | 11 | 0,00001 |
| ssc-miR-532-5p | 45 | 52 | 52 | 38 | 57 | 52 | 0,00003 |
| ssc-miR-423-3p | 44 | 63 | 71 | 52 | 104 | 39 | 0,00004 |
| ssc-miR-22-5p | 43 | 8 | 1 | 50 | 7 | 1 | 0,00001 |
| ssc-miR-499-5p | 40 | 32 | 30 | 37 | 63 | 24 | 0,00002 |
| ssc-miR-342 | 37 | 34 | 58 | 56 | 67 | 39 | 0,00003 |
| ssc-miR-1306-5p | 36 | 43 | 40 | 45 | 66 | 30 | 0,00003 |
| ssc-miR-28-3p | 36 | 55 | 51 | 31 | 79 | 47 | 0,00003 |
| ssc-miR-500 | 32 | 34 | 26 | 21 | 43 | 23 | 0,00002 |
| ssc-miR-7 | 31 | 39 | 41 | 23 | 50 | 45 | 0,00002 |
| ssc-miR-101 | 26 | 24 | 26 | 22 | 27 | 18 | 0,00001 |
| ssc-miR-16 | 26 | 35 | 17 | 8 | 29 | 30 | 0,00001 |
| ssc-miR-148b | 25 | 161 | 83 | 36 | 255 | 68 | 0,00006 |
| ssc-miR-532-3p | 22 | 21 | 19 | 14 | 38 | 21 | 0,00001 |
| ssc-miR-92a | 22 | 26 | 34 | 17 | 49 | 17 | 0,00002 |
| ssc-miR-17-3p | 22 | 3 | 3 | 57 | 3 | 7 | 0,00001 |
| ssc-miR-339-5p | 21 | 14 | 15 | 19 | 22 | 9 | 0,00001 |
| ssc-miR-130b | 20 | 43 | 25 | 44 | 101 | 17 | 0,00003 |
| ssc-miR-365-3p | 19 | 31 | 21 | 23 | 71 | 10 | 0,00002 |
| ssc-miR-17-5p | 18 | 11 | 16 | 39 | 26 | 11 | 0,00001 |
| ssc-miR-128 | 16 | 10 | 10 | 16 | 17 | 12 | 0,00001 |
| ssc-miR-99a | 16 | 37 | 47 | 22 | 40 | 28 | 0,00002 |
| ssc-miR-151-3p | 15 | 21 | 40 | 10 | 27 | 20 | 0,00001 |
| ssc-miR-361-3p | 13 | 6 | 6 | 11 | 15 | 3 | 0,00001 |
| ssc-miR-423-5p | 12 | 12 | 20 | 21 | 22 | 16 | 0,00001 |
| ssc-miR-22-3p | 12 | 15 | 45 | 15 | 27 | 25 | 0,00001 |
| ssc-miR-664-5p | 12 | 5 | 15 | 15 | 9 | 5 | 0,00001 |
| ssc-miR-374a* | 11 | 14 | 13 | 11 | 16 | 6 | 0,00001 |
| ssc-miR-34a | 11 | 18 | 5 | 10 | 46 | 1 | 0,00001 |
| ssc-miR-30a-5p | 10 | 9 | 18 | 10 | 7 | 7 | 0,00001 |
| ssc-miR-125a | 10 | 9 | 25 | 7 | 47 | 41 | 0,00001 |
| ssc-miR-424* | 9 | 1 | 1 | 7 | 0 | 1 | 0,00000 |
| ssc-miR-542-3p | 9 | 14 | 5 | 2 | 5 | 3 | 0,00000 |
| ssc-miR-210 | 9 | 11 | 18 | 16 | 10 | 11 | 0,00001 |
| ssc-miR-450b-5p | 8 | 19 | 2 | 0 | 1 | 1 | 0,00000 |
| ssc-miR-95 | 8 | 14 | 11 | 8 | 14 | 11 | 0,00001 |
| ssc-miR-10a | 8 | 10 | 10 | 5 | 15 | 8 | 0,00001 |
| ssc-miR-628 | 7 | 2 | 15 | 11 | 8 | 6 | 0,00000 |
| ssc-miR-92b-3p | 7 | 12 | 7 | 4 | 6 | 2 | 0,00000 |
| ssc-miR-99b | 7 | 2 | 1 | 2 | 18 | 5 | 0,00000 |
| ssc-miR-152 | 7 | 3 | 4 | 8 | 4 | 5 | 0,00000 |
| ssc-miR-505 | 6 | 12 | 6 | 20 | 23 | 7 | 0,00001 |
| ssc-miR-32 | 6 | 7 | 7 | 2 | 2 | 3 | 0,00000 |
| ssc-miR-424 | 6 | 27 | 9 | 9 | 6 | 3 | 0,00001 |
| ssc-miR-19b | 5 | 4 | 1 | 7 | 7 | 1 | 0,00000 |
| ssc-miR-425-3p | 5 | 5 | 13 | 6 | 7 | 10 | 0,00000 |
| ssc-miR-361-5p | 5 | 17 | 5 | 2 | 13 | 7 | 0,00000 |
| ssc-miR-450c-5p | 5 | 3 | 7 | 0 | 0 | 1 | 0,00000 |
| ssc-miR-455 | 4 | 15 | 3 | 11 | 10 | 4 | 0,00000 |
| ssc-miR-126 | 4 | 2 | 5 | 2 | 1 | 1 | 0,00000 |
| ssc-miR-193a-5p | 4 | 0 | 4 | 5 | 2 | 8 | 0,00000 |
| ssc-miR-195 | 4 | 8 | 10 | 5 | 35 | 12 | 0,00001 |
| ssc-miR-574 | 3 | 6 | 19 | 5 | 19 | 17 | 0,00001 |
| ssc-miR-1839-5p | 3 | 2 | 1 | 0 | 1 | 1 | 0,00000 |
| ssc-miR-30b-3p | 3 | 0 | 1 | 4 | 2 | 0 | 0,00000 |
| ssc-miR-140 | 3 | 2 | 7 | 3 | 7 | 4 | 0,00000 |
| ssc-miR-497 | 3 | 2 | 5 | 3 | 3 | 5 | 0,00000 |
| ssc-miR-331-5p | 3 | 3 | 6 | 8 | 2 | 5 | 0,00000 |
| ssc-miR-331-3p | 3 | 7 | 2 | 9 | 30 | 4 | 0,00001 |
| ssc-miR-133a-3p | 2 | 3 | 1 | 0 | 6 | 1 | 0,00000 |
| ssc-miR-202 | 2 | 0 | 1 | 3 | 0 | 2 | 0,00000 |
| ssc-miR-328 | 2 | 3 | 11 | 2 | 3 | 7 | 0,00000 |
| ssc-miR-1307 | 2 | 7 | 8 | 4 | 11 | 6 | 0,00000 |
| ssc-miR-324 | 2 | 10 | 8 | 10 | 28 | 7 | 0,00001 |
| ssc-miR-92b-5p | 1 | 0 | 0 | 0 | 0 | 0 | 0,00000 |
| ssc-miR-185 | 1 | 7 | 3 | 3 | 4 | 3 | 0,00000 |
| ssc-miR-421 | 1 | 0 | 1 | 0 | 0 | 1 | 0,00000 |
| ssc-miR-151-5p | 1 | 6 | 2 | 1 | 14 | 2 | 0,00000 |
| ssc-miR-205 | 1 | 0 | 0 | 0 | 1 | 1 | 0,00000 |
| ssc-miR-181c | 1 | 1 | 0 | 1 | 1 | 0 | 0,00000 |
| ssc-miR-374b-3p | 1 | 1 | 3 | 2 | 1 | 0 | 0,00000 |
| ssc-miR-107 | 1 | 1 | 3 | 0 | 3 | 0 | 0,00000 |
| ssc-miR-133b | 1 | 1 | 0 | 0 | 4 | 1 | 0,00000 |
| ssc-miR-183 | 1 | 8 | 15 | 8 | 74 | 47 | 0,00002 |
| ssc-miR-192 | 1 | 6 | 11 | 27 | 5 | 43 | 0,00001 |
| ssc-miR-345-3p | 1 | 0 | 0 | 0 | 1 | 0 | 0,00000 |
| ssc-miR-542-5p | 1 | 0 | 0 | 0 | 0 | 1 | 0,00000 |
| ssc-miR-126* | 0 | 1 | 0 | 0 | 0 | 0 | 0,00000 |
| ssc-miR-34c | 0 | 4 | 7 | 3 | 2 | 1 | 0,00000 |
| ssc-miR-215 | 0 | 2 | 5 | 0 | 0 | 0 | 0,00000 |
| ssc-miR-193a-3p | 0 | 0 | 1 | 0 | 1 | 1 | 0,00000 |
| ssc-miR-181b | 0 | 0 | 0 | 0 | 1 | 0 | 0,00000 |
| ssc-miR-199b | 0 | 0 | 1 | 0 | 0 | 0 | 0,00000 |
| ssc-miR-450a | 0 | 0 | 1 | 0 | 1 | 2 | 0,00000 |
| ssc-miR-204 | 0 | 1 | 0 | 1 | 0 | 0 | 0,00000 |
| ssc-miR-365-5p | 0 | 0 | 3 | 0 | 1 | 0 | 0,00000 |
| ssc-miR-106a | 0 | 0 | 0 | 3 | 2 | 0 | 0,00000 |
| ssc-miR-1839-3p | 0 | 0 | 0 | 0 | 0 | 1 | 0,00000 |
| ssc-miR-30a-3p | 0 | 2 | 4 | 1 | 2 | 0 | 0,00000 |
| ssc-miR-451 | 0 | 1 | 3 | 1 | 2 | 0 | 0,00000 |
| ssc-miR-1277 | 0 | 0 | 1 | 0 | 0 | 1 | 0,00000 |
| ssc-miR-143-3p | 0 | 0 | 1 | 0 | 0 | 0 | 0,00000 |
| ssc-miR-504 | 0 | 0 | 0 | 1 | 0 | 0 | 0,00000 |
| ssc-miR-708-3p | 0 | 1 | 0 | 0 | 0 | 0 | 0,00000 |
| ssc-miR-708-5p | 0 | 0 | 2 | 0 | 0 | 0 | 0,00000 |
| ssc-miR-129 | 0 | 0 | 0 | 0 | 0 | 2 | 0,00000 |
| ssc-miR-28-5p | 0 | 2 | 1 | 2 | 9 | 2 | 0,00000 |
| ssc-miR-139-5p | 0 | 1 | 0 | 11 | 1 | 0 | 0,00000 |
| ssc-miR-326 | 0 | 2 | 1 | 0 | 1 | 0 | 0,00000 |
| ssc-miR-335 | 0 | 1 | 0 | 0 | 1 | 0 | 0,00000 |
| ssc-miR-4336 | 0 | 0 | 0 | 0 | 0 | 1 | 0,00000 |
| ssc-miR-664-3p | 0 | 3 | 1 | 1 | 2 | 0 | 0,00000 |
| ssc-miR-181d-5p | 0 | 1 | 0 | 0 | 2 | 0 | 0,00000 |
| ssc-miR-196b-5p | 0 | 1 | 0 | 0 | 0 | 0 | 0,00000 |
| ssc-miR-676-3p | 0 | 0 | 0 | 0 | 0 | 1 | 0,00000 |
| ssc-miR-155 | 0 | 0 | 1 | 1 | 3 | 0 | 0,00000 |
| ssc-miR-133a-5p | 0 | 0 | 3 | 0 | 7 | 1 | 0,00000 |
| ssc-miR-100 | 0 | 1 | 0 | 0 | 0 | 0 | 0,00000 |
| ssc-miR-181a | 0 | 0 | 1 | 0 | 0 | 0 | 0,00000 |
