# Supplementary material for: Co-Expression of Host and Viral MicroRNAs in Porcine Dendritic Cells Infected by the Pseudorabies Virus
Source: PLoS One. 2011 Mar 8;6(3):e17374. doi: 10.1371/journal.pone.0017374 (PMC3050891; doi:10.1371/journal.pone.0017374)
Supplement: Table S3 — Newly predicted swine miRNAs in DCs. Mature sequence and sequence read count of new porcine miRNAs predicted with miRDeep in each sequenced sample of DCs (mock-infected: GDI3, GDI5, GDI7; infected by PRV: GDI4, GDI6, GDI8). In the first part of the table miRNA 21–24 nt long are listed, while the second part shows miRNA of less common length (<21 or >24). (DOC) [file pone.0017374.s006.doc]

### Supporting Table 3. Newly predicted swine miRNAs in DCs.

Mature sequence and sequence read count of new porcine miRNAs predicted with miRDeep in each sequenced sample of DCs (mock-infected: GDI3, GDI5, GDI7; infected by PRV: GDI4, GDI6, GDI8). In the first part of the table miRNA 21-24 nt long are listed, while the second part shows miRNA of less common length (<21 or >24).

| **mature seq (21-24 nt long)** | **GDI3** | **GDI4** | **GDI5** | **GDI6** | **GDI7** | **GDI8** |
| --- | --- | --- | --- | --- | --- | --- |
| ACAGATGATGAACTTATTGACG | - | - | - | - | 135 | - |
| ATCATGTATGATACTGCAAAC | - | 6 | - | - | 26 | - |
| ATGCATCTTTCCCTGGATTTAGG | - | - | - | - | 2 | - |
| ATTGTTCTCCAACCTGGCTCT | - | - | - | - | 4 | - |
| CCATTCCTTCGTCTGTGCACTAGA | - | 4 | - | - | - | - |
| GAGAGATCAGAGGCGCAGAGT | 147 | 172 | 227 | 220 | 273 | 304 |
| TAGGGCACAGGATGGGATGAGGA | - | - | - | - | - | 8 |
| TCGACCGGACCTCGACCGGCT | - | - | - | - | 25 | - |
| TCTAGCATCGAGCACCCGCCT | 28 | 27 | 42 | 54 | 30 | 19 |
| TCTGAGATGTGACCTGGGCAT | 53 | 30 | 22 | 53 | 61 | 16 |
| TGTTGTACTTTTTTTTTTGTT | - | - | 13 | - | - | - |
| TTGGTGGGGACATGGTGCTTCT | 14 | - | - | - | - | - |
| TTTACGTCCTTTCACCTAGTTT | - | - | 3 | - | - | - |
| TTTGCTCTGCTCCTGCCACATGC | - | - | 31 | 14 | 19 | 17 |
| **mature seq (< 21 and >24 nt long)** | |  |  |  |  |  |
| AGCTGTGGTGTAGGTCA | - | 2 | - | - | - | - |
| AGGAGGGAGGAGATGGGCCAAGTTC | - | - | 15 | - | - | - |
| AGGATTCCTGGTTTTCA | - | - | - | - | - | 13 |
| AGGGAGAGAACGCGGTCTGAGTGGT | - | - | - | - | - | 55 |
| ATTGGTGGTTCAGTGGTAGAATTCTC | - | 355 | - | - | - | - |
| CAGCATGTTTCCAAGGG | - | - | - | - | 49 | - |
| CCCGCCTCCTCTCGCCG | - | - | 15 | - | 14 | - |
| CCCGGCCAACGCACCA | - | - | 11 | - | - | 27 |
| GGATTCCTGGTTTTCA | - | 20 |  | 12 | 25 | 13 |
| GGGGTGGGGGTCTGGGGG | 19 | - | - | - | - | - |
| GTGCCAGGTGGGGAGTTTGA | 43 | - | - | - | - | - |
| TCCCGGCCAACGCACCA | - | - | - | - | - | 27 |
| TGGTTAGTACTTGGATGG | - | - | - | - | 55 | - |
